# Participatory Strategies to Enhance Resilience and Job Satisfaction and Reduce Stress to Mitigate Early Retirement Intentions Among Nurses: Protocol for a Qualitative Study

**DOI:** 10.2196/72089

**Published:** 2025-06-30

**Authors:** Ghada Derbel, Alexandra Lecours

**Affiliations:** 1 Département d’ergothérapie Université du Québec à Trois-Rivières Trois-Rivières, QC Canada; 2 Center for Interdisciplinary Research in Rehabilitation and Social Integration Québec, QC Canada

**Keywords:** occupational stress, resilience, job satisfaction, early retirement, nurses, qualitative research, protocol

## Abstract

**Background:**

As Canada’s population ages, so does its workforce. Early retirement among nurses is on the rise and has become the norm within this workforce. It represents a major concern for maintaining an adequate and qualified workforce. On the one hand, the decision to take early retirement can be influenced by various factors, including occupational stress. By contrast, low job satisfaction can exacerbate early retirement intentions, while resilience is positively associated with the intention to remain at work. Little is known about how to mobilize these factors to promote healthy job retention for nurses as they age.

**Objective:**

This study aims to (1) explore the experiences of older nursing staff regarding their intention to take early retirement and the influence of occupational stress, resilience, and job satisfaction; (2) explore interventions used to optimize the influence of resilience and job satisfaction and minimize the influence of occupational stress on early retirement; and (3) generate and validate participatory strategies tailored to the context of older nursing staff to optimize the influence of resilience and job satisfaction and minimize the influence of occupational stress on early retirement.

**Methods:**

A 3-phase qualitative research design will be used. In phase 1, we will use an interpretive descriptive design using semistructured interviews to explore the experience surrounding early retirement intentions and related factors among nurses. In phase 2, we will use a scoping review to explore interventions used to optimize the influence of resilience and job satisfaction and minimize the influence of occupational stress on early retirement. In phase 3, we will use the technique for research of information by animation of a group of experts method with a group of 8 participants. This method will allow us to generate and validate participatory strategies tailored to the context of older nurses.

**Results:**

Initial results are expected in August 2025. The findings of this study will be shared through multiple platforms to maximize their reach and impact. This will include publishing scientific articles, completing a research dissertation, and presenting at conferences. A concise summary document highlighting key findings will be sent to study participants, who will also have the option to receive links to the online publications derived from the research.

**Conclusions:**

This protocol presents detailed information about the entire structure of the 3-phase research project. Studying early retirement issues among older nurses is essential. It promotes their health, retention, and inclusion, and recognizes their contributions to the sector.

**International Registered Report Identifier (IRRID):**

PRR1-10.2196/72089

## Introduction

### Background

The aging of Canada’s population has become a predominant demographic feature [1]. In Canada, between 1921 and 2018, the median age increased by approximately 16 years, from 23.9 to 40.8 years, and it will continue to increase steadily until 2034 [1]. By 2043, 24.3% of the Canadian population will be aged >65 years [1]. With the aging of the population comes the aging of its workforce [2]. An older worker is defined as a worker who is aged ≥55 years [3]. In 1996, there were 2.7 Canadian workers aged 25 to 34 years for every worker aged ≥55 years. By 2018, this ratio had decreased to 1 [1]. This ratio is insufficient to offset the declining number of younger workers [4], a situation that could be attributed to declining birth rates as well as the increasing number of young adults pursuing higher education [5,6].

In the same context, workers who provide care to an increasingly older population are aging themselves [2]. Among Canadian nurses, approximately 1 in 5 were aged ≥55 years in 2016, compared to <1 in 10 in 1996 [2]. As nurses age, they retire, and issues such as labor shortages and the loss of the most experienced care staff members arise [7]. Considering the difficult working conditions of nurses, such as long hours and staff shortages, this can increase the tendency to retire relatively early [8]. In 2001, the average retirement age of nurses in Canada was 56 years [9]. With the aging of this workforce, this average has slightly increased to 58.1 years in 2019 [10], but it remains relatively early compared to the average retirement age in the general population in Canada, which is 64.4 years [11]. In addition, the distinct challenges faced by this population may increase the risk of early retirement. Nurses are considered to be a susceptible population as it is composed mostly of older, female individuals from an immigrant background [12-14]. Advancing age is not without health risks, as older workers are more susceptible to both physical and psychosocial issues, which can lead to periods of disability, whether related to occupational injuries or personal health conditions such as chronic illness [15]. Some studies suggest that female nurses are more frequently exposed to verbal violence compared to their male counterparts [16], while others have reported that female health care providers face a higher prevalence of violence, particularly sexual violence [17,18]. Immigrant nurses face unique challenges that place them at a disadvantage in the workplace. Studies have shown that immigrants are more often exposed to social exclusion [19]. Particularly, immigrant nurses must navigate complex cultural and organizational adjustments, which can lead to higher levels of work-related stress and ultimately increase their likelihood of leaving the profession or retiring early [20]. Moreover, the discrimination and marginalization of internationally educated nurses in host countries are well documented, often exacerbated by communication barriers, which can further hinder their ability to thrive in the workplace [21].

Early retirement is usually defined according to age. In the province of Québec in Canada, workers in the public sector are eligible for early retirement when they are aged 55 years [22].

Early retirement is a major concern when it comes to maintaining a sufficient, qualified workforce [23]. According to the World Health Organization, the shortage of health care professionals reached 7.2 million worldwide in 2013 [24], and this number will reach 18 million by 2030 [25]. Nursing staff shortage leads to a low caregiver-to-patient ratio [26]. It is also associated with stress, burnout, and job dissatisfaction among nurses [26,27].

In this context, there are several factors influencing the decision of these nurses to take early retirement. We have grouped the influencing factors in terms of individual, family, and occupational factors [28]. Individual factors can include the health condition of workers and their economic status [29]. Family factors may include the marital status of older nurses, in which studies show that those with spouses retire early [13,30]. Occupational factors can refer to the work environment factors [31], such as occupational stress, workload, decision latitude, and satisfaction with the work environment [13,30,32-35].

In this regard, occupational stress among nurses increases the risk of early retirement [36]. Occupational stress is defined as the harmful physical and emotional responses that occur when work demands do not match the worker’s abilities, resources, or needs [37]. Nurses are constantly exposed to occupational stress, which increased during the COVID-19 pandemic, becoming a real occupational hazard [38,39]. In addition, low job satisfaction would exacerbate intentions to take early retirement among older nursing staff [34]. Job satisfaction is defined as a general feeling about the job or a large set of related attitudes about various aspects or facets of the job [40]. Low job satisfaction is one of the most important factors in the increase in early retirement intentions [41,42]. It is influenced by several factors, such as heavy workloads and long working hours [43], and it further increases occupational stress in these workers [44], which further increases the risk of early retirement.

By contrast, there are factors that can decrease the intention of early retirement, namely resilience, which is linked to the intention to stay at work and is a protective factor for personal health [44]. Resilience is defined as the ability to recover from adversity, uncertainty, failure, or even positive changes that seem overwhelming, such as increased responsibility [45]. Resilience in older nursing staff can reduce occupational stress, increase job satisfaction [44], and negatively influence intentions to take early retirement [46].

In view of the increasing number of older nurses and the problem of early retirement and labor shortage among nurses, it is important to study the reality of older nurses and to find strategies in order to counter the problem of early retirement among this population.

### Aim

The general aim of this study is to promote the health and job retention of older nurses through the creation of participatory strategies by examining the influence of resilience, job satisfaction, and occupational stress on early retirement.

## Methods

### Overview

**Figure 1:**
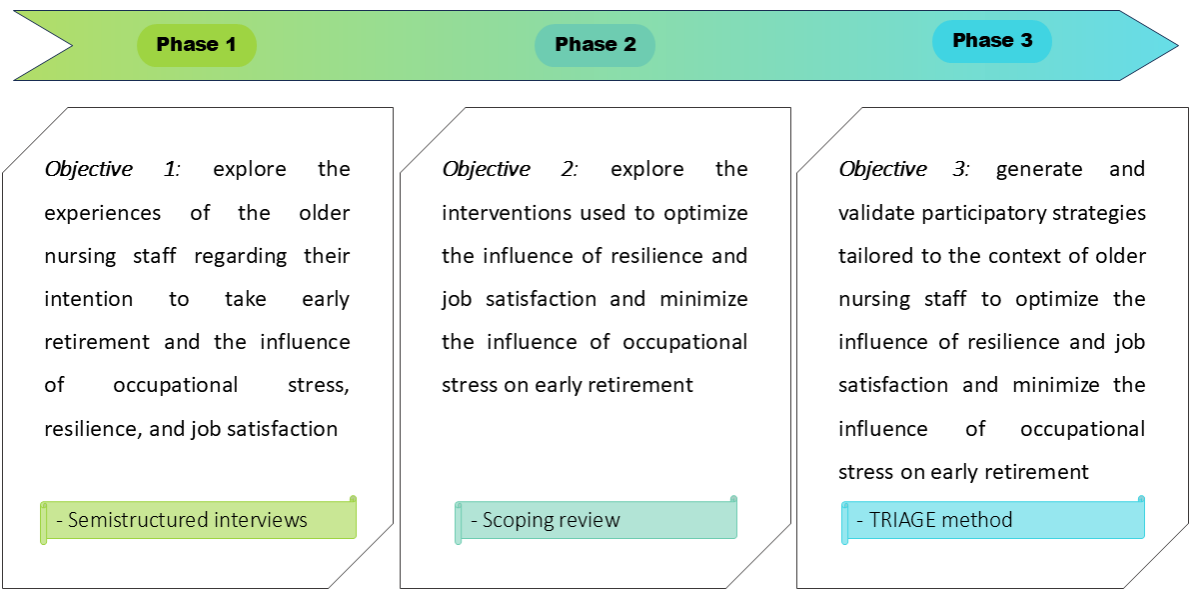
Research approach. TRIAGE: technique for research of information by animation of a group of experts.

To reach our aim, a 3-phase qualitative study will be conducted. The first phase will explore the experiences of older nurses, their views on or intentions of retiring early, and the influence of occupational stress, resilience, and job satisfaction. The second phase will explore the interventions used to optimize the influence of resilience and job satisfaction and minimize the influence of occupational stress on early retirement. In the third phase, we will generate and validate participatory strategies tailored to the context of older nurses to optimize the influence of resilience and job satisfaction and minimize the influence of occupational stress on early retirement. Figure 1 shows our research approach.

### Phase 1

#### Design

To describe the experiences of older nurses and their intentions to retire early, a descriptive interpretative study will be conducted [47].

#### Population

This study will focus on nursing personnel working in the public sector (ie, registered nurses, nurse practitioners, clinicians, and licensed practical nurses). To be eligible for participation, individuals would have to be employed as a nurse in the public sector and possess ≥5 years of professional experience. The literature indicates that a minimum of 3 to 5 years of work experience is necessary to effectively examine workers’ attitudes toward employment and career decisions [48,49]. Furthermore, participants must fall within the age range of 45 to 55 years. Our chosen age group is deliberate, as individuals in our target population become eligible for early retirement when they are aged 55 years. Consequently, we are focusing on a slightly younger demographic because this study will be centered on examining the intention to retire early. We will strive to target diversity among our participants in terms of gender, cultural and ethnic backgrounds, and practice settings (eg, general and specialist care, mental health, and perinatal care) to ensure our sample reflects various perspectives within the studied population. However, this diversity is framed within a homogeneous sample in terms of profession (nursing staff) and professional context (public sector), which will allow us to deeply explore the shared experiences and perceptions of this older population regarding early retirement.

#### Sampling and Recruitment

An initial number of 20 participants is estimated for this study. This number is based on findings from previous research [50,51]. For example, some authors report that when participants belong to a relatively homogeneous group, as is the case in our study, where all participants are nursing staff working in the public health care sector, data saturation can generally be achieved with ≤16 interviews [50]. Other authors suggest that 6 to 12 participants may be sufficient to reach saturation when the group shares a similar reality, such as working in the same field [52]. In contrast, samples with greater heterogeneity may require >20 participants to achieve saturation [50,51]. While our sample may present some diversity in terms of practice settings, it remains professionally and contextually homogeneous. Therefore, setting an initial target of 20 participants strikes a balance between ensuring diversity within a focused context and achieving thematic saturation. Saturation will be reached, and recruitment will stop when redundancy is observed in the ideas put forward by participants [52-54]. The final number will be adjusted during the course of the study based on the percentage of new information identified in each interview.

Using the saturation assessment method developed by Guest et al [55], we will agree on the final number of participants to recruit in order to achieve data saturation at a threshold of 95%. Thus, based on the initial sample size set at 20 and a new information threshold set at 5%, we will continue conducting interviews until they yield <5% new information. At that point, recruitment will end, as we will have reached 95% saturation.

Participant recruitment will be done using a nonprobabilistic convenience method on a voluntary basis [54,56]. We will use a recruitment poster to invite potential participants. A digital version of the poster will be published on the *Chaire de recherche UQTR (jr) sur la santé des travailleuses et travailleurs vieillissants* (Research Chair on the Health of Aging Workers) website and its LinkedIn page as well as shared via other social media platforms. Members of the research team will also disseminate the poster via their personal networks. In addition, the Université du Québec à Trois-Rivières (UQTR) Partnership Service will be requested to share the poster within its network of collaborators. To further enhance outreach, the poster will be sent to union organizations and nursing associations across the province. We will also contact the various nursing orders in Québec to request access to their email distribution lists. A formal procedure will be established, which includes completing a request form and submitting the information and consent forms to the relevant professional orders for nurses. We will also use snowball sampling by encouraging participants to share the study information with their colleagues who may be eligible and interested in participating.

Participation in this study is entirely voluntary. Participants are completely free to participate or to withdraw at any time without penalty and without having to provide any explanation. If a participant decides to withdraw from the study, all data provided will be deleted and will not be used as part of this research.

#### Data Collection

We will conduct semistructured interviews based on a guide developed from a comprehensive review of the literature on early retirement, occupational stress, job satisfaction, and resilience among nurses. The guide will then be pretested with 2 people having the same characteristics as our participants [57]. We will document various themes (eg, intention to retire early, influence of occupational stress, satisfaction, and resilience) by asking different questions (eg, “What are your current thoughts on early retirement?” “How has the stress you experience at work influenced your thoughts on early retirement?” “How has your job satisfaction evolved throughout your career?” and “Can you share any key experiences or moments?”) [54]. Interviews will be conducted either in person or online via Microsoft Teams, depending on the participant’s choice. If a participant opts for an in-person interview, it will take place in a familiar and comfortable location of their choice. In both cases, the interview will be audio recorded to allow for an in-depth qualitative analysis. Each interview is expected to last approximately 90 minutes to allow for an in-depth exploration of the research topics [58,59]. However, participants will be informed of the expected duration in advance and will be free to take comfort breaks at any time during the interview. They will also be reminded that they can stop the interview at any moment without providing any justification. The interview process will be flexible and respectful of each participant’s pace and comfort. Should any participant experience emotional distress during or after the interview, appropriate support resources will be made available. Participants will receive information on local social services and community organizations that offer emotional and psychological support. Public sector employees may also benefit from support and representation offered by local unions, such as the *Fédération de la Santé et des Services Sociaux* in cases of work-related concerns. Moreover, participants will have access to the *Employee Assistance Program*, a free and confidential professional support service provided to staff and managers to help address personal or professional challenges. Although these resources will be provided to participants, we cannot guarantee that all participants will be eligible for or able to access every service.

#### Data Analysis Plan

The first author will transcribe the interviews. When interview recordings have been fully transcribed verbatim, a qualitative data analysis will be conducted using a five-step thematic analysis strategy: (1) repeated reading of the data corpus to allow analysts to develop a sense of immersion, (2) initial coding of identified units of meaning, (3) assigning meaningful labels to the coded units of meaning, (4) synthesizing and assembling codes into a general structure with categories and themes, and (5) “moving back and forth” between the data corpus and the general structure to ensure the interpretation of selected units [60]. Two analysts will independently analyze each interview. Afterward, they will meet after each analysis to discuss, compare, and integrate their coding to generate a common version that ensures interjudge agreement [61,62]. Subsequently, a third person will review the coding of each interview to provide input on the coherence of ideas. We will adopt an inductive approach in this thematic analysis. This approach is well suited for descriptive interpretive research [47]. NVivo software (version 15; Lumivero) will be used to support the analysis.

### Phase 2

#### Design

To explore the interventions used to optimize the influence of resilience and job satisfaction and minimize the influence of occupational stress on early retirement, we will opt for a scoping review design [63]. This choice is motivated by the need to comprehensively examine the amount, range, and nature of existing research related to a specific topic, namely the interventions designed to decrease early retirement intentions [63,64]. To uphold scientific rigor throughout our review process, we will adhere to the PRISMA-ScR (Preferred Reporting Items for Systematic Reviews and Meta-Analyses Extension for Scoping Reviews) guidelines [65]. These guidelines are specifically designed for scoping reviews and will serve as our framework for transparently reporting the insights derived from our scoping review [65].

#### Procedure and Analysis

The review followed the Joanna Briggs Institute (JBI) methodology for scoping reviews, with further refinements made in accordance with the guidelines outlined by Peters et al [66].

#### Step 1: Review Question

The research question should be broad and exploratory to include the most relevant manuscripts [64]. A clear scoping review question informs the inclusion criteria, supports protocol development, enhances the efficiency of the literature search, and provides a structured foundation for the review [66]. The main research question that will guide the scoping review is as follows: What interventions are used to optimize the influence of resilience and job satisfaction and minimize the influence of occupational stress on early retirement?

#### Step 2: Inclusion Criteria

Manuscripts will be selected based on the following inclusion criteria: (1) manuscripts addressing interventions regarding resilience, job satisfaction, occupational stress, or any combination of the 3 and (2) manuscripts addressing the context of work. All types of manuscripts are eligible, with the exception of literature review–type studies, which will be considered only to examine the studies they include, as this will avoid duplicate representation of studies and allow us to add any unretrieved articles meeting the selection criteria [67]. The inclusion of all types of manuscripts, with the exception of literature reviews, will allow capturing the full breadth of available evidence on interventions related to resilience, job satisfaction, and occupational stress. It will allow us to comprehensively examine the amount, range, and nature of existing research related to the interventions designed to decrease early retirement intentions, which aligns with best practices recommended for the conduct of scoping reviews [64,68]. While this approach may result in the inclusion of manuscripts that are less rigorous or interventions that are less relevant, selection steps (ie, step 4) will be strict and function as additional filters. The selection will be conducted based on the predefined inclusion and exclusion criteria as well as the relevance of each manuscript to the research objective, ensuring that only pertinent data are included in the study. The inclusion criteria will be tested on a sample of 5 selected manuscripts to ensure clarity, and adjustments will be made if necessary.

To provide a contemporary picture of the situation, only manuscripts published within the last 15 years will be selected. The decision to limit the search to studies published within the last 15 years is made in accordance with the JBI methodology, which emphasizes the importance of using date restrictions in an informed manner, as potentially relevant studies may be missed if the time set is too recent [69]. The JBI methodology also emphasizes that the search strategy for a scoping review should aim to be as comprehensive as possible within the constraints of time and resources [66]. In response to that and while the health care system has undergone changes over time [70], interventions developed in the past 15 years may remain relevant and continue to be used or adapted in present-day practice. For feasibility reasons, only manuscripts written in English and French will be included.

#### Step 3: Search Strategy

A subject specialist librarian will be consulted to generate the search strategy used for the scoping review [71]. Different combinations of keywords (eg, interventions AND resilience) will be generated. Because the subject covers several areas of interest (ie, health, gerontology, and management), we will conduct systematic searches on different databases selected based on their relevance to the main areas of interest (eg, APA PsycINFO [EBSCO], CINAHL [EBSCO], MEDLINE [EBSCO], AMED [EBSCO], Scopus, and ABI/INFORM Global).

To ensure the most comprehensive search for identifying manuscripts, we will adopt a strategy involving data retrieval from various sources. In doing so, we will also review the reference lists of selected manuscripts to identify other relevant manuscripts.

Furthermore, gray literature documents will be examined by manually searching on Google. Due to the large number of results generated by Google searches, it is important to rely on the relevancy ranking that brings the most relevant results to the first pages. Hence, the titles and short descriptions found in the first 10 pages will be screened [72]. Targeted searches on the websites of relevant health care organizations and public health databases will be conducted (eg, the *Institut de recherche Robert-Sauvé en santé et en sécurité du travail* and the *Institut national de santé publique du Québec*).

#### Step 4: Source of Evidence Selection

Following the search, all identified manuscripts will be uploaded to EndNote (Clarivate) software.

Then, selection will be performed by 2 reviewers, with a third reviewer intervening in case of disagreement on manuscript eligibility. A 2-step approach will be used for selection. First, reviewers will examine the title and abstract to determine eligible manuscripts. Second, the full texts of selected manuscripts will be examined by the 2 reviewers.

#### Step 5: Data Extraction

Data extraction will be carried out using an extraction grid based on the TIDieR (Template for Intervention Description and Replication) checklist, with several criteria to be followed (eg, intervention name, who provided the intervention, what materials and procedures were used in the intervention, where did the intervention occur, why is the intervention conducted, and how is the intervention delivered) [73], to ensure the comprehensiveness of information on interventions used to optimize the influence of resilience and job satisfaction and minimize the influence of occupational stress on early retirement. Such a grid has previously been found useful to describe interventions in the field of work [74]. The grid will first be tested by 2 reviewers by independently extracting data from 25% of the manuscripts. Following a meeting between the 2 reviewers, a final version of the grid will be presented and used for extraction without modification.

#### Step 6: Data Analysis and Presentation

This final step involves presenting the results by providing a general overview of the literature and identifying knowledge gaps [66]. This step will include a description of the selected manuscripts and a narrative summary of the results [66]. First, a descriptive analysis using statistics will be used to present a descriptive numerical summary of the extent, nature, and distribution of the studies, such as the distribution of studies geographically, and the range of interventions [66]. Next, a thematic analysis will be conducted to group the interventions extracted from the selected manuscripts into key themes, such as resilience-building strategies, improving job satisfaction, and occupational stress management [66]. The steps of thematic analysis will follow the same steps that will be used in phase 1.

### Phase 3

#### Design

We will use the technique for research of information by animation of a group of experts (TRIAGE) [75] to generate and validate participatory strategies tailored to the context of older nurses. These strategies aim to optimize the influence of resilience and job satisfaction and minimize the influence of occupational stress on early retirement. This method aligns with the participatory approach adopted for phase 3 of this project, as it provides a user-friendly way to engage individuals affected by a common issue [76]. The TRIAGE method is chosen as it allows for consensus building on a specific topic through participant discussions while fostering the emergence of new ideas [77]. This process will help generate and achieve consensus on various participatory strategies adapted to the context of older nurses to optimize the influence of resilience and job satisfaction and minimize the influence of occupational stress on early retirement.

#### Population

According to Tétreault and Guillez [78], TRIAGE groups with 6 to 8 participants are often the most productive. Our TRIAGE group will consist of 8 participants, including nursing staff. The inclusion criteria are the same as those of phase 1. The recruitment methods for this phase will mirror those used in phase 1, namely a nonprobabilistic convenience sampling approach based on voluntary participation, supplemented by snowball sampling. In addition, participants from phase 1 will be contacted to assess their interest in taking part in this subsequent phase.

#### Data Collection

##### Overview

**Figure 2:**
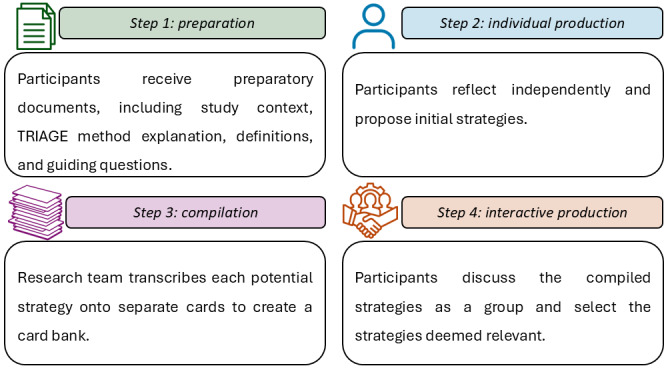
The technique for research of information by animation of a group of experts (TRIAGE) method.

The TRIAGE method will involve four systematic steps: (1) preparation, (2) individual production, (3) compilation, and (4) interactive production. Figure 2 illustrates the steps of the TRIAGE method.

##### Step 1: Preparation

Before the individual production, we will provide participants with preparatory documents containing information about the study’s context and the TRIAGE method. The preparatory documents will be prepared by the research team. This includes the study’s objectives, key concept definitions, a brief description of the TRIAGE method, and a summary of the results from phases 1 and 2 [77]. In addition, we will develop questions to guide participants’ strategy proposals (eg, How to create a less stressful work environment to encourage older nurses to stay at work?). Questions will concern early retirement and our 3 factors of interest (ie, occupational stress, job satisfaction, and resilience). These documents will be sent electronically and pretested with 2 individuals matching the participants’ characteristics to ensure content relevance and clarity [75].

##### Step 2: Individual Production

During this individual step, participants (ie, older nurses) will generate participatory strategies tailored to the context of older nurses. To do so, they will be asked to answer the questions developed during step 1 independently and remotely, in a setting of their choice, by suggesting potential strategies [75,76]. This step will start once participants receive the preparatory documents and the questions electronically. Participants will be given 2 weeks to respond to the questions and return them to the research team.

##### Step 3: Compilation

This step will be conducted by the research team. It will involve transcribing each potential strategy generated in step 2 onto separate cards without modification or analysis [75]. This will create a card bank containing potential strategies for each question (ie, for each of our 3 factors of interest regarding early retirement). Each card bank will be processed separately during the interactive production step [77].

##### Step 4: Interactive Production

This step will be characterized by the animation of a sorting process with visual support, illustrating six sections: (1) dynamic memory, (2) grouping, (3) selection, (4) garbage bin, (5) refrigerator, and (6) veto [75,76]. This step will be conducted through an online group meeting via Microsoft Teams involving the first author and the participants.

First, all potential strategies will be displayed in the *dynamic memory* section. Once participants understand all the potential strategies, they will transfer the potential strategies they deem relevant to the *grouping* section and those they deem irrelevant to the *garbage bin* section [75]. The latter will be permanently discarded [77]. Next, the potential strategies transferred to the *grouping* section will be discussed. Those with similar meanings will be merged and given more precise titles. Potential strategies containing more than one idea may be split into distinct parts, each with new titles [77]. Discussion in this section may also lead to the emergence of new potential strategies [75].

In case of uncertainty among participants, a potential strategy will be temporarily placed in the *refrigerator* section for later reconsideration to avoid prolonging momentarily unproductive discussions [77]. This will allow the sorting process to continue and promote group harmony [77]. Ideally, if there is still disagreement, a possible strategy will be placed in the *veto* section to be submitted to other experts at a later date [77]. Finally, the potential strategies definitively placed in the *selection* section will constitute the participatory strategies retained in this study [77].

This step will last a maximum of 3 hours to ensure data quality. This time frame will enable the participants to offer pertinent information, while extending beyond this time frame will risk compromising results due to increased participant and animator fatigue [77].

#### Data Analysis Plan

The participatory strategies collected using the TRIAGE method will be immediately validated through group interactions, eliminating the need for verbatim transcription and content analysis [75]. In other words, rapid data analysis will help validate participatory strategies tailored to the context of older nurses, optimizing the influence of resilience and job satisfaction and minimizing the influence of occupational stress on early retirement.

### Ethical Considerations

An information and consent form detailing the objectives of the study, the modalities of participation, the duration of the participation, and the process of the individual interviews will be sent to the participant by email before the interview date in phase 1 and before step 1 (preparation) in phase 3. The participant will have the opportunity to read the form carefully and will provide written consent. Financial compensation will be provided to each participant after the completion of the interview to compensate for any expenses incurred through their participation, such as the time devoted to the interview and any other personal constraints related to their participation.

Each participant will receive financial compensation upon completion of the TRIAGE group discussions, in recognition of any expenses incurred through their participation, including the time devoted to the study and any personal constraints associated with their involvement.

To ensure data confidentiality and participant anonymity, a coding system will be used for the interviews. All data will be kept for a period of 5 years after the conclusion of the study and then securely destroyed to ensure confidentiality and data protection. They will not be used for purposes outside the scope of this research. Finally, all the information written and recorded during the various stages of the study (such as interviews and transcripts) will be kept in a computerized, password-protected file and a secure OneDrive (Microsoft Corporation) file. Ethics approval from the human research ethics committee of the Université du Québec à Trois-Rivières has been obtained (CER-24-313-07.07).

## Results

Initial results are expected in August 2025. This study aspires to promote the health and job retention of older nurses through the creation of participatory strategies by examining the influence of resilience, job satisfaction, and occupational stress on early retirement.

On the basis of the results, actionable recommendations for both practice and policy will be formulated.

The findings of this study will be disseminated through various channels to maximize their impact and ensure they reach the intended audience. This will include the publication of scientific articles, the completion of a research dissertation, and participation in conferences through presentations. In addition, a concise summary document highlighting the main research findings will be prepared and emailed to the participants. They will also have the option to receive a link to the online publications resulting from the study.

## Discussion

### Anticipated Findings

The aim of this study is to promote the health and job retention of older nurses through the creation of participatory strategies by examining the influence of resilience, job satisfaction, and occupational stress on early retirement. The results of this study could make numerous contributions to the advancement of knowledge and provide input for practice, organization, society, and decision makers.

For practice, the results of this study will support the proposal of concrete strategies to foster a healthier work environment for nurses. These authors recognize the importance of finding systemic strategies dedicated to health care workers, especially after their experiences throughout the COVID-19 pandemic [44]. For instance, studies reveal a positive influence of intervention programs based on optimizing resilience, which results in lower stress and lower burnout symptoms among nurses [78,79]. Consequently, this promotes the health of these workers and promotes a healthy work environment. In fact, having a healthy work environment for older nurses is very important for several reasons. First, it influences the staff’s perception of feeling supported and appreciated at work [80]. Second, it has a reducing effect on the shortage of nursing staff members [81]. Finally, it helps nursing staff provide effective, quality patient care and improve the health care system [27,82]. On another note, while conducting the study, the participatory approach in the development of these strategies can help older nurses feel more integrated and part of a community at work. In fact, having a sense of community in which workers consider their place of work as a resource for meeting key social and psychological needs, such as the need for inclusion and affiliation, is found to be a major predictor of job satisfaction [83]. Although the strategies proposed because of this study will have to be assessed for their realism in the light of organizational and societal realities, the fact that they will emerge from the workers themselves will optimize their probability of success. The intention of the study is that the findings will inform not only academic knowledge but also the development of workplace interventions tailored to support older nurses; therefore, the next step will prioritize engagement with health care stakeholders to facilitate the implementation and evaluation of these strategies in future research as well as into organizational policies and support programs.

On a societal and an organizational level, early retirement is a major problem for labor shortages [7]. By addressing this problem, this study would be promoting the job retention of the most experienced workers, thereby mitigating labor shortages. Decision makers, specifically the employers of nurses, are faced with a very worrying problem that is turnover, and one of the top reasons for nurses’ turnover is retirement [84]. In the United States, nurses’ turnover costs are at an all-time high, as the average cost of turnover for a nurse can be >US $50,000, resulting in the average hospital losing between US $6.6 million and US $10.5 million [85]. With >1 million nurses projected to retire between 2020 and 2030 [86], retaining these older workers is essential to the sustainability of the workforce. Consequently, having tailored strategies for older nurses, which are available to their employers, would be a breakthrough, as it would allow them to effectively benefit from reduced turnover rates due to retirement, which, in turn, lowers recruitment and training costs. In this sense, strategies to support nurses’ health and well-being must not merely be short-term and discrete initiatives; employers must embed them systematically into every aspect of nursing, including retirement [87]. However, it is worth mentioning that these strategies must be complemented with a restructuring of organizational processes to ensure their effectiveness [88]. For instance, if the strategy proposed is based on promoting meditation for nurses to minimize occupational stress during their shifts, but the employers discourage nurses from taking breaks, then the effectiveness of this strategy is likely to be considerably diminished. Although this is a process involving many actors and factors, the results of this study will provide some initial information for reflection on such a process.

### Strengths and Limitations

In the context of this study, we will generate participatory strategies based on increasing resilience and job satisfaction and decreasing occupational stress to reduce early retirement intentions among nurses. By including this population in the process of developing strategies, we can gain a better understanding of the unique needs, preferences, and challenges faced by this specific population and tailor strategies to address them. Furthermore, the rigor and transparency in the methodological process will allow for adequate reproducibility.

On the other hand, while scoping reviews promote a broad overview of a specific topic, they do not seek to assess the quality of evidence [63]. Consequently, we cannot determine whether the studies selected for the review will provide robust or generalizable findings, and we cannot ensure the effectiveness of all interventions.

For the purpose of this study, only nurses will be invited to participate in the TRIAGE phase of the study, as the objective is to generate strategies directly from their lived experiences and perspectives. This approach will ensure that the strategies will be tailored to their specific needs and realities. Moreover, maintaining a homogenous group of participants (all being nurses) will support group cohesion and encourage more open dialogue during the collective production process, even if they come from diverse practice settings [77]. However, it is important to acknowledge that this approach does not capture the perspectives of other key stakeholders, such as nurse managers or decision makers, whose input could also be relevant in the development and implementation of workplace strategies. Exploring their views could be the focus of a subsequent study to complement and enrich the findings of this study.

### Conclusions

Health care services face a number of challenges, including an older workforce, early retirement, and labor shortages. Through this study, we will explore, in depth, the factors pushing older nurses toward early retirement and develop strategies to modify them. These workers are undoubtedly an invaluable source of experience for the health care sector. Therefore, it becomes imperative to not only retain them in the workforce but also ensure an environment that will sustain their well-being. The care sector will benefit from the skills and knowledge of older nurses, which will help alleviate the labor shortages.

In conclusion, this study seeks to inform the development of participatory strategies that may support resilience, enhance job satisfaction, and mitigate occupational stress among older nurses. While the effectiveness of these strategies remains to be empirically evaluated, the findings are expected to offer valuable insights that could guide future initiatives addressing similar challenges across the health care sector.

## Abbreviations

JBI: Joanna Briggs Institute
PRISMA-ScR: Preferred Reporting Items for Systematic Reviews and Meta-Analyses Extension for Scoping Reviews
TIDieR: Template for Intervention Description and Replication
TRIAGE: technique for research of information by animation of a group of experts
UQTR: Université du Québec à Trois-Rivières
